# Various myosteatosis selection criteria and their value in the assessment of short- and long-term outcomes following liver transplantation

**DOI:** 10.1038/s41598-021-92798-5

**Published:** 2021-06-28

**Authors:** Franziska Alexandra Meister, Jan Bednarsch, Iakovos Amygdalos, Joerg Boecker, Pavel Strnad, Philipp Bruners, Sven Arke Lang, Tom Florian Ulmer, Lara Heij, Daniel Antonio Morales Santana, Wen-Jia Liu, Georg Lurje, Ulf Peter Neumann, Zoltan Czigany

**Affiliations:** 1 Department of Surgery and Transplantation, Faculty of Medicine, University Hospital RWTH Aachen, Pauwelsstrasse 30, 52074 Aachen, Germany; 2 Department of Internal Medicine III, Faculty of Medicine, University Hospital RWTH Aachen, Aachen, Germany; 3 Institute of Radiology, Faculty of Medicine, University Hospital RWTH Aachen, Aachen, Germany; 4 Institute for Pathology, Faculty of Medicine, University Hospital RWTH Aachen, Aachen, Germany; 5 Department of Surgery, Campus Charité Mitte | Campus Virchow-Klinikum, Charité-Universitätsmedizin Berlin, Berlin, Germany; 6 Department of Surgery, Maastricht University Medical Centre (MUMC), Maastricht, The Netherlands

**Keywords:** Medical research, Outcomes research

## Abstract

Body composition and myosteatosis affect clinical outcomes in orthotopic liver transplantation (OLT). Here we aimed to compare the value and limitations of various selection criteria to define pre-transplant myosteatosis in the assessment of short- and long-term outcomes following OLT. We retrospectively analyzed the data of 264 consecutive recipients who underwent deceased donor OLT at a German university medical centre. Myosteatosis was evaluated by preoperative computed-tomography-based segmentation. Patients were stratified using muscle radiation attenuation of the whole muscle area (L3Muslce-RA), psoas RA (L3Psoas-RA) and intramuscular adipose tissue content (IMAC) values. L3Muslce-RA, L3Psoas-RA and IMAC performed well without major differences and identified patients at risk for inferior outcomes in the group analysis. Quartile-based analyses, receiver operating characteristic curve and correlation analyses showed a superior association of L3Muslce-RA with perioperative outcomes when compared to L3Psoas-RA and L3IMAC. Long-term outcome did not show any major differences between the used selection criteria. This study confirms the prognostic role of myosteatosis in OLT with a particularly strong value in the perioperative phase. Although, based on our data, L3Muscle-RA might be the most suitable and recommended selection criterion to assess CT-based myosteatosis when compared to L3Psoas-RA and L3IMAC, further studies are warranted to validate these findings.

## Introduction

While body composition (BC) may strongly vary among individuals, the generalized loss of muscle mass, function and strength defined as sarcopenia is frequently observed in critically ill patients^1^. A progressive sarcopenia is an underappreciated and frequent complication in patients with end stage liver disease (ESLD) and can be present in 40 to 60% of the patient undergoing orthotopic liver transplantation (OLT)^2^. Over the past ten years, an increasing number of reports demonstrated that the status of the skeletal muscle compartment has a significant prognostic value in various oncological and chronic diseases. Previous studies have shown the association of sarcopenia with inferior waiting list and post-transplant outcomes^3–6^. Excessive pathological intramuscular fat disposition called “myosteatosis” has recently been independently correlated with an increased risk of inferior outcomes in cancer and in end-stage liver disease^1,7,8^. Recent studies by our group have identified not only a high prevalence of myosteatosis but a strong association with adverse perioperative outcomes in patients undergoing OLT^4^.

Although, various techniques (e.g. dual-energy X-ray absorptiometry, magnetic resonance imaging, bioimpedance analysis) are used to assess patient BC in the clinical setting, cross-section imaging studies, such as computed tomography (CT), are recognized by the recent Clinical Practice Guidelines of the European Association for the Study of the Liver (EASL) as the gold standard for the quantification of clinically significant structural alteration in the skeletal muscle compartment^9^. Muscle mass (sarcopenia) and quality (myosteatosis) are usually estimated by segmentation of the cross-sectional area at the level of third lumbar vertebra (L3). Even though, myosteatosis is typically defined by low muscle radiation attenuation (RA) values in Hounsfield units (HU), there are multiple selection criteria introduced by different groups to characterize myosteatosis and identify patient at risk, without a clear international consensus^4,5,10^. Frequently utilized are the absolute values of muscle attenuation using sex-specific cutoffs of the whole skeletal muscle area (including psoas major, erector spinae, quadratus lumborum, transversus abdominis, external and internal obliques, and rectus abdominis) versus the bilateral psoas muscle area^4,10^. A novel selection criterion for the assessment of skeletal muscle quality and myosteatosis has been first described by Kitajima in non-alcoholic fatty liver disease and further explored by Hamaguchi et al. in the setting of living donor liver transplantation^5,11,12^. Intramuscular adipose tissue content or IMAC is defined as the lumbar multifidus muscle / subcutaneous fat tissue attenuation ratio^5,12^. Despite the fact that all of the above described muscle attenuation or myosteatosis selection criteria have been used in various patient cohorts, there is no clinical data directly comparing their value in the prediction of post-transplant outcomes in the setting of deceased donor OLT.

In this study we aimed to comprehensively assess the performance of three frequently used selection criteria for myosteatosis (L3Muscle-RA; L3Psoas-RA; L3IMAC) in predicting post-transplant outcomes in a large European single-center cohort of adult patients undergoing deceased donor OLT.

## Patients and methods

### Patients and eligibility

**Figure 1:**
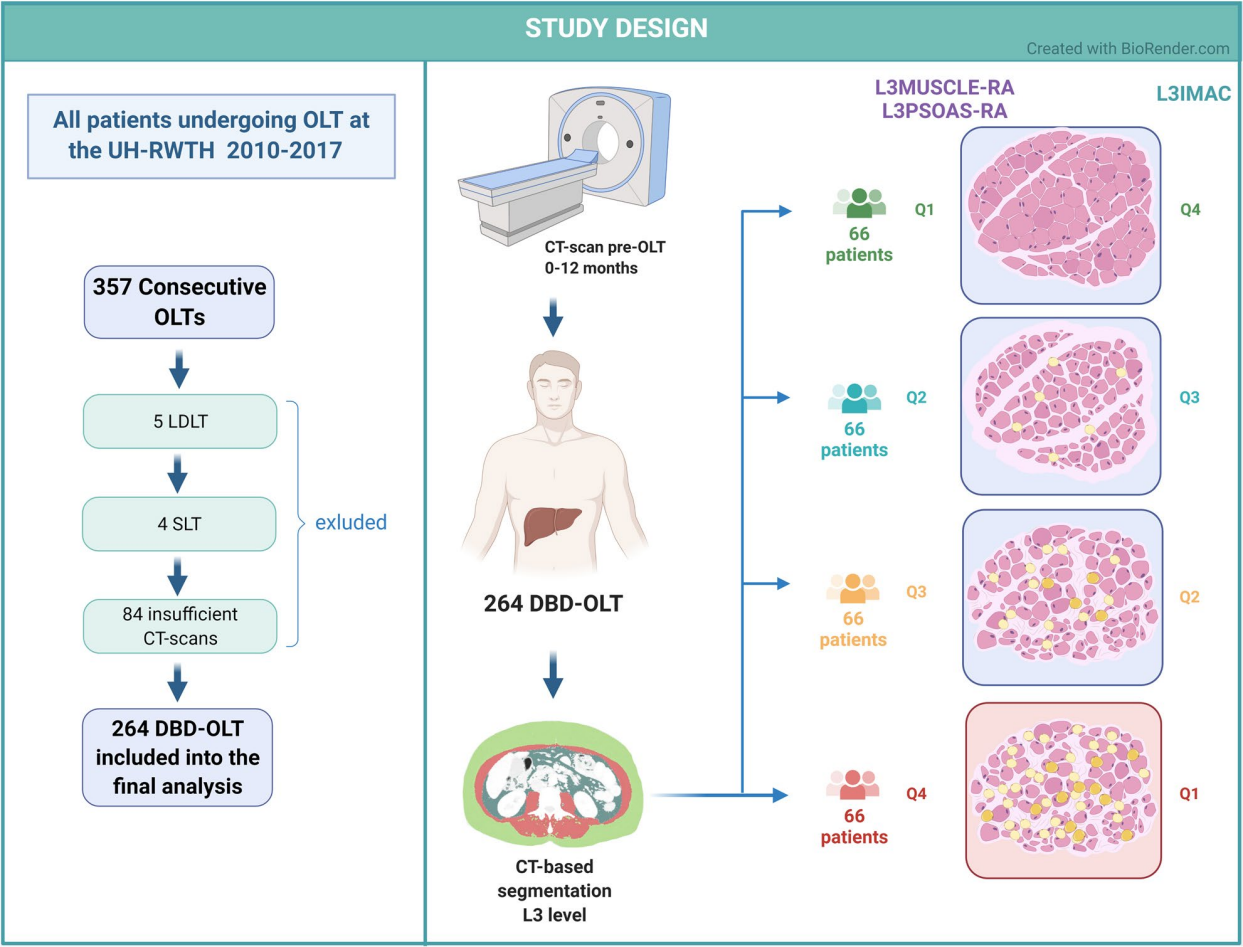
Study flowchart and design. The figure was created with BioRender.com (www.biorender.com). *Abbreviations used*: CT: computed tomography; OLT: orthotopic liver transplantation; UH-RWTH: University Hospital of the RWTH University; LDLT: living donor liver transplantation; SLT: split liver transplantation; DBD: donation after brain death, L3: lumbar 3, L3Muscle-RA: lumbar 3 muscle radiation attenuation, L3Psoas-RA: lumbar 3 Psoas radiation attenuation, L3IMAC: lumbar 3 intramuscular adipose tissue content, Q: quartile.

All consecutive OLT recipients undergoing liver transplantation between 05/2010 and 12/2017 at the University Hospital RWTH Aachen (UH-RWTH), Aachen, Germany, were considered for inclusion into this retrospective analysis (Fig. 1). Although all patients have received a CT imaging prior to OLT, patients with insufficient imaging (L3 level not included and/or CT scan older than 12 months) were not eligible for the study. Recipients of living-related or deceased donor split liver transplantation were also excluded.

### Ethics and informed consent statement

This study followed the principles of the current version of the Declaration of Helsinki as well as the Declaration of Istanbul, and the good clinical practice (ICH-GCP) guidelines and was approved by the RWTH-Aachen University Institutional Review Board (EK 047/18). Informed consent was waived by the IRB (“Ethik-Kommission der RWTH Aachen”) due to the retrospective study design and collection of routine clinical data.

### Image analysis

Computed tomography imaging and CT segmentation were carried out as described before^4^. Briefly, image data of the most recent preoperative CT-scan were analyzed by the same investigator who was blinded for the remaining clinical data and outcomes of the patients. A single cross-sectional image/patient has been analyzed at the level of the third lumbar vertebra using the 3D Slicer software platform version 4.1 and BC module (https://www.slicer.org/) as described before^4,13,14^. Table 1 shows the definitions and attenuation cutoff values used in the segmentation analysis. Sex- and cohort-specific cutoff values have been defined by the first and last quartiles of the corresponding body composition parameters (Q1 for intramuscular adipose tissue content-IMAC; Q4 for L3Psoas-Radiation Attenuation (RA) and for L3Muscle-RA; see Table 1 and Fig. 1) as recently described by Kalafateli et al^15^. Sex-specific (male–female) cutoff values are important to correct for the gender-associated differences in muscle density and volume.

**Table 1:** Cut-off values and body composition parameters.

Body composition	Area and definition^b^	Interpretation	Patient cutoff values^c^		Mean (± SD)/median [IQR]	
			Female	Male	Female	Male
L3Muscle-RA (HU)	L3 whole skeletal muscle area	Decreasing values indicate more low attenuation myosteatotic muscle, thus inferior muscle quality	< 26.6	< 28.6	34.0 [26.6–41.1]	37.8 [28.6–43.5]
L3Psoas-RA (HU)	L3 bilateral psoas area	Decreasing values indicate more low attenuation myosteatotic muscle, thus inferior muscle quality (similar to L3Muscle-RA)	< 38.9	< 40.0	44.4 ± 8.3	45.2 ± 8.6
L3IMAC (HU)^**a**^	L3 region of interest (ROI) of the multifidus muscle/ROI of subcutaneous fat	Increasing values indicate more low attenuation myosteatotic muscle, thus inferior muscle quality	> − 0.32	> − 0.35	− 0.41 ± 0.14	− 0.43 ± 0.13

*Abbreviations used*: L3Muscle-RA: lumbar 3 muscle radiation attenuation, HU: Hounsfield units, L3Psoas-RA: lumbar 3 Psoas radiation attenuation, L3IMAC: lumbar 3 intramuscular adipose tissue content, SD: standard deviation; IQR, interquartile range.

^a^Based on Kitajima et al. and Hamaguchi et al.^5,40^.

^b^Following attenuation cutoff values were used to differentiate between various tissue components during image analysis according to literature definitions: Muscle: − 29 to 150 HU, Visceral adipose tissue: − 150 to − 50 HU, Subcutaneous adipose tissue: − 190 to − 30.

^c^Patient cutoff values were determined based on the sex- and cohort-specific distribution of L3Muscle-RA and L3Psoas-RA (last quartile—Q4) as well as IMAC values (first quartile—Q1) to identify patients at risk of inferior outcomes. Values were given as median and [interquartile range-IQR] or mean ± standard deviation in case of normal distribution.

### Clinical data collection and patient follow up

Clinical data were recovered from a prospective institutional database, medical charts and analyzed in a retrospective fashion. Liver allocation followed German national and international Eurotransplant regulations. The liver transplantation procedure was performed using a standardized approach of total cava replacement as previously described^16–18^. Perioperative treatment and immunosuppression were performed in a standardized fashion as described before^16,17^. The RWTH Aachen transplantation outpatient clinic and the responsible community-based hepatologists provided the follow-up data used in this study.

All definitions, scores and classifications used in this manuscript have been described by our group and by others in previous reports (including OLT risk scores^19–22^, definitions of extended criteria donor allografts-ECD and early allograft dysfunction-EAD^23–25^, indications for OLT listing^26^, Clavien-Dindo classification-CD and the Comprehensive Complication Index-CCI^27,28^, calculations of the length of ICU and hospital stay^29^, procedural costs^30^, peri- and postoperative transfusions^4^).

### Statistical analysis

The primary endpoint of the present study was the incidence of 90-day post-OLT major morbidity (defined by CD ≥ 3b)^27^. Overall perioperative outcome, length of ICU- and hospital stay, mortality, EAD, procedural costs, long-term graft- and recipient survival were analyzed and reported as secondary endpoints.

Normal distribution was tested using the Kolmogorov–Smirnov-test for continuous variables. Data was reported as mean and standard deviation for normally distributed, and median (interquartile range-IQR) was displayed for non-normally distributed data. Absolute and relative frequencies were reported in case of categorical variables. For the statistical comparison of continuous variables, the Student t test, the Mann–Whitney U test, and the Kruskal–Wallis H test were used where appropriate. The Chi-square test and the Fisher’s exact test were used, for the analysis of categorical data. To determine the ability of myosteatosis to predict perioperative outcome, uni- and multivariable logistic regression analyses were performed. Spearman correlation coefficient was used to further analyze the association of various clinical outcomes and myosteatosis. The further discriminative ability of the various myosteatosis selection criteria for the prediction of outcomes was compared using the receiver operating characteristic (ROC) analysis calculating the area under the receiver operating characteristic curve (AUROC). The Hosmer–Lemeshow Chi^2^ goodness-of-fit test was applied to test model suitability. The level of statistical significance was defined as *p* < 0.05 and the statistical analysis has been performed using SPSS Statistics v24 (IBM Corp., Armonk, NY, USA).

## Results

### Study population and characteristics

Of the 357 consecutive patients who underwent OLT within the given study period, 84 had no sufficient preoperative CT imaging including the L3 level within the last 12 months before OLT, 5 patients underwent living related and 4 split liver transplantations. The exclusion of these patients resulted in a final study cohort of 264 patients with a median donor and recipient age of 56 [47–66] and 55 [48–61] years, respectively (Table 2). The median interval between the CT scan used for segmentation and OLT was 6 [2–19] weeks. Some 165 patients (66%) were male. The Kolmogorov–Smirnov-test showed a non-normal distribution for all analyzed variables with the exception of L3Psoas-RA and L3IMAC (*p* = 0.200; *p* = 0.200).

**Table 2:** Donor and recipient characteristics.

Characteristics	n = 264
**Donor characteristics**	
Donor sex (F:M)	123 (47%) : 141 (53%)
Donor age (years)	56 [47–66]
Donor BMI	28 [25–31]
Donor Risk Index^a^	1.77 [1.51–2.02]
Donor cause of death	CVA 164 (62%) Anoxia 55 (21%) Trauma 31 (12%) Other 14 (5%)
Extended Criteria Donor Allografts^b^	173 (66%)
**Recipient characteristics**	
Recipient sex (F:M)	89 (34%) : 175 (66%)
Recipient age (years)	55 [48–61]
Recipient BMI	26 [23–31]
Listing Indication	ALF 34 (13%) HCC 73 (28%) Alcoholic cirrhosis 54 (21%) Viral 18 (7%) PSC/PBC 25 (9%) Graft failure 4 (1%) NASH 14 (5%) Other 45 (17%)
Pre-transplant Child–Pugh Score	7 [5–9]
Pre-transplant labMELD	17 [10–27]
BAR Score^c^	8 [4–13]
SOFT Score^d^	11 [8–18]
Recipient pre-transplant ICU	62 (24%)
Recipient pre-transplant abdominal surgery	94 (36%)
Recipient pre-transplant encephalopathy	101 (38%)
Cold ischemic time (min)	482 [426–577]
Warm ischemic time (min)	45 [40–50]
Intra-operative platelet transfusions (units)	0 [0–2]
Intra-operative red blood cell transfusions (units)	7 [4–11]
Intra-operative fresh frozen plasma transfusions (units)	15 [12–20]
Post-operative platelet transfusions (units)^e^	0 [0–1]
Post-operative red blood cell transfusions (units)^e^	2 [0–4]
Post-operative fresh frozen plasma transfusions (units)^e^	2 [0–8]

Values were given as median and (interquartile range-IQR) or numbers and (per cent).

*Abbreviations used*: POD: postoperative day, BMI: body mass index, DRI: donor risk index, CVA: cerebrovascular accident, ECD: extended criteria donor allografts, ALF: acute liver failure, HCC: hepatocellular carcinoma, PSC: primary sclerosing cholangitis, PBC: primary biliary cholangitis, AIH: autoimmune hepatitis, MELD: model for end-stage liver disease, PLT: platelet, BAR: balance of risk, SOFT: survival outcomes following liver transplantation, CCI: comprehensive complication index, ICU: intensive care unit.

^a^Based on Feng et al.^42^.

^b^Based on the German Medical Chamber Guidelines^31^.

^c^Based on Schlegel et al.^43^.

^d^Based on Rana et al.^21^.

^e^Defined as blood products given during the first 7-days following OLT.

The most common indications for OLT were hepatocellular carcinoma (28%) and alcoholic liver cirrhosis (21%). In compliance with the German law on organ donation, all donors were donors after brain death (DBD), with cerebrovascular accidents (62%) as the leading cause of death, followed by anoxia (21%) and trauma (12%). Sixty-six percent (173) of the transplanted liver allografts fulfilled the ECD criteria for DBD donors^31^. The median pre-transplant laboratory MELD score of the cohort was 17 [10–27]. Detailed donor and recipient characteristics are displayed in Table 2.

### Distribution of body composition parameters and sex-specific cutoff values

**Figure 2:**
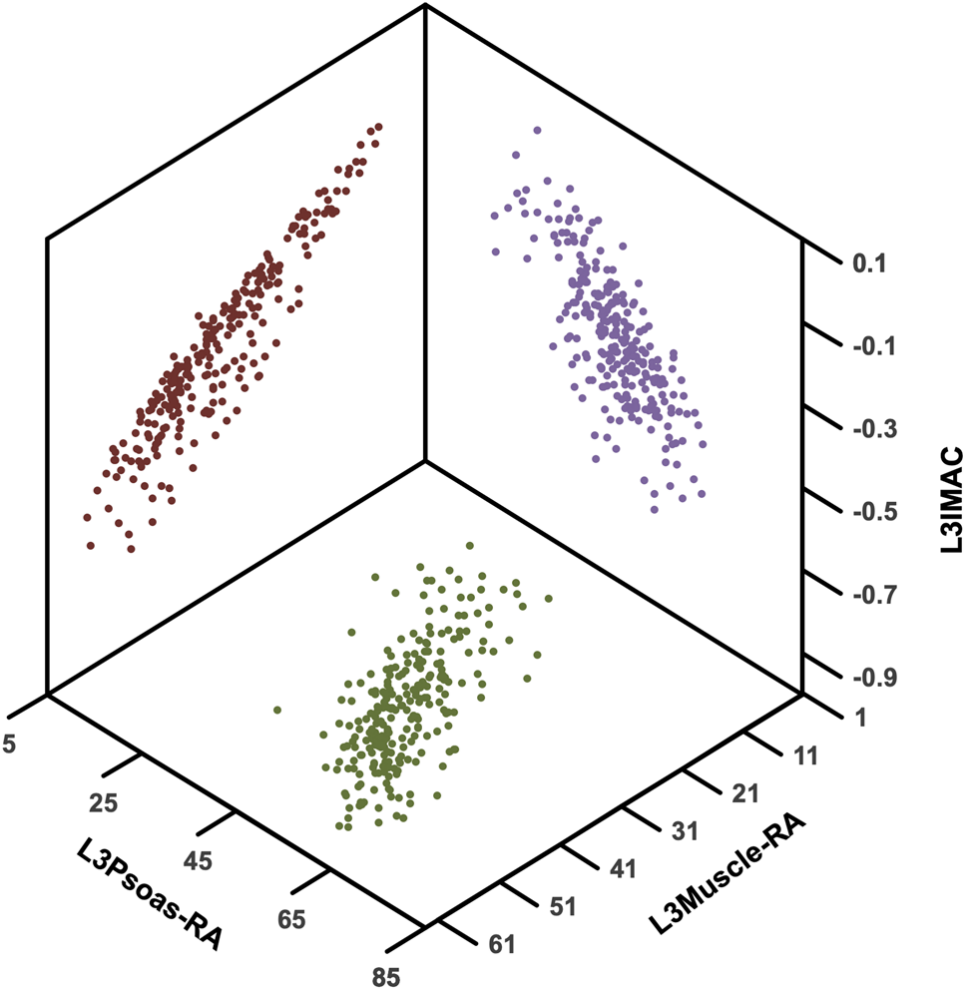
3D scatter plot showing the association between L3Muscle-RA, L3Psoas-RA, L3IMAC. The Spearman correlation coefficient showed a moderate to strong correlation between the various selection criteria. L3Muscle-RA/L3Psoas: r = 0.776 *p* < 0.0001; L3Muscle-RA/L3IMAC: r = − 0.768 *p* < 0.0001; L3Psoas-RA/L3IMAC: r = − 0.546 *p* < 0.0001. *Abbreviations used*: L3Muscle-RA: lumbar 3 muscle radiation attenuation, L3Psoas-RA: lumbar 3 Psoas radiation attenuation, L3IMAC: lumbar 3 intramuscular adipose tissue content.

The median L3Muscle-RA was 37.8 [28.6–43.5] HU for male and 34 [26.6–41.1] HU for female recipients. The mean L3Psoas-RA was 45.2 ± 8.6 HU for male and 44.4 ± 8.3 HU for female patients and the mean L3IMAC was − 0.43 ± 0.13 HU for male and − 0.41 ± 0.14 HU for female patients, respectively (Table 1). The Spearman correlation coefficient showed a moderate to strong correlation between the used selection criteria (Fig. 2). L3Muscle-RA/L3Psoas: r = 0.776 *p* < 0.0001; L3Muscle-RA/L3IMAC: r = − 0.768 *p* < 0.0001; L3Psoas-RA/L3IMAC: r = − 0.546 *p* < 0.0001.

Body composition profiles calculated from the segmentation of the preoperative CT scans and the stratification of the OLT patient cohort based on the sex-specific quartile-based cut-off values are summarized in Table 1.

### Perioperative outcomes

52% (136 out of 264) of all recipients developed major (CD ≥ 3b) post-transplant complications within the first 90-day following OLT according to the definitions of the Clavien–Dindo classification (Table 3). The median cumulative CCI score for the patient cohort was 50 [30–80], and OLT recipients spent a median of 5 [3–10] days on ICU and 27 [20–47] days in hospital, respectively (Table 3).

**Table 3:** Group analysis of perioperative outcome based on the L3Muscle-RA, L3Psoas-RA and L3IMAC cutoffs.

	All patients	No	Yes	*p* value
**Myosteatosis L3Muscle-RA**	n = 264	n = 198	n = 66	
90-day ≥ CD3b complications^a^ n (%)	136 (52)	88 (45)	47 (72)	0.000
90-day mortality n (%)	20 (8)	9 (5)	11 (18)	0.002
Early allograft dysfunction^b^n (%)	72 (28)	48 (24)	24 (36)	0.016
ICU stay (days)	5 [3–10]	4 [3–8]	7 [5–29]	0.000
Hospital stay (days)	27 [20–47]	24 [20–40]	42 [24–80]	0.000
Intraoperative RBC transfusion (units)	7 [4–11]	6 [4–10]	10 [7–15]	0.000
Intraoperative FFP transfusion (units)	15 [12–20]	15 [10–20]	16 [12–23]	0.092
90-day CCI^c^	50 [30–80]	46 [24–65]	70 [45–100]	0.000
Cost estimation (TEuro)^d^	52 [39–76]	49 [36–63]	68 [47–106]	0.000
**Myosteatosis L3Psoas-RA**	n = 264	n = 198	n = 66	
90-day ≥ CD3b complications n (%)	136 (52)	90 (46)	45 (68)	0.001
90-day mortality n (%)	20 (8)	10 (5)	10 (15)	0.008
Early allograft dysfunction n (%)	72 (28)	52 (26)	20 (30)	0.279
ICU stay (days)	5 [3–10]	4 [3–8]	7 [4–22]	0.000
Hospital stay (days)	27 [20–47]	24 [19–41]	38 [24–75]	0.000
Intraoperative RBC transfusion (units)	7 [4–11]	6 [4–10]	9 [7–14]	0.000
Intraoperative FFP transfusion (units)	15 [12–20]	15 [10–20]	16 [12–22]	0.106
90-day CCI	50 [30–80]	47 [26–67]	63 [43–99]	0.000
Cost estimation (TEuro)	52 [39–76]	49 [37–66]	60 [47–99]	0.000
**Myosteatosis L3IMAC**	n = 264	n = 198	N = 66	
90-day ≥ CD3b complications n (%)	136 (52)	91 (46)	44 (67)	0.000
90-day mortality n (%)	20 (8)	9 (5)	11 (17)	0.001
Early allograft dysfunction n (%)	72 (28)	48 (24)	24 (36)	0.016
ICU stay (days)	5 [3–10]	4 [3–8]	8 [5–30]	0.000
Hospital stay (days)	27 [20–47]	25 [20–43]	37 [22–75]	0.003
Intraoperative RBC transfusion (units)	7 [4–11]	6 [4–10]	10 [7–16]	0.000
Intraoperative FFP transfusion (units)	15 [12–20]	15 [10–20]	16 [14–25]	0.006
90-day CCI	50 [30–80]	47 [24–66]	70 [44–100]	0.000
Cost estimation (TEuro)	52 [39–76]	49 [36–64]	68 [47–107]	0.000

*Abbreviations used*: L3Muscle-RA: lumbar 3 muscle radiation attenuation, CD: Clavien–Dindo classification, ICU: intensive care unit, RBC: red blood cell units, FFP: fresh frozen plasma units, CCI: Comprehensive Complication Index, TEuro: thousand Euros, L3Psoas-RA: lumbar 3 psoas radiation attenuation, L3IMAC: lumbar 3 intramuscular adipose tissue content.

^a^Based on Clavien et al.^27^.

^b^Based on Olthoff et al.^23^.

^c^Based on Slankamenac et al.^28^.

^d^Based on Staiger et al.^30^.

The overall incidence of EAD was 72 out of 264 (27%). A median of 7 [4–11] RBC and 15 [12–20] FFP units were administered intraoperatively and the median estimated procedural costs over the first 90-days were 52 [39–76] TEuro (Table 3).

### L3Muscle-RA, L3Psoas-RA and L3IMAC as myosteatosis selection criteria and their value in predicting perioperative outcomes

First, we analyzed the suitability of the three myosteatosis selection criteria to stratify our patient cohort into high and low-risk groups based on morbidity and mortality using sex-specific cutoff values (Q1 for IMAC; Q4 for L3Muscle-RA and L3Psoas-RA; see Table 1 and Fig. 1).

As shown in Table 3, subgroups of patients beyond the cut-off values of L3Muscle-RA, L3Psoas-RA and L3IMAC had significantly more major complications (71% vs. 45%, 68% vs. 46%, 67% vs. 46%; *p* < 0.001, *p* < 0.01, *p* < 0.001 for CD ≥ 3b, respectively; see Table 3), higher 90-day mortality (18% vs. 5%, 15% vs. 5%, 17% vs. 5%, *p* < 0.01, *p* < 0.01, *p* < 0.001, respectively; see Table 3) and higher 90-day cumulative CCI scores (70 [45–100] vs. 46 [24–65], 63 [43–99] vs. 47 [26–67], 70 [44–100] vs. 47 [24–66], *p* < 0.001, for all, Table 3). All three myosteatosis selection criteria showed a comparable performance and identified patients at risk for long ICU (7 [5–29] vs. 4 [3–8] days, 7 [4–22] vs. 4 [3–8] days, 8 [5–30]. 4 [3–8] days, *p* < 0.001, for all, Table 3) and total in-hospital stay (42 [24–80] vs. 24 [20–40] days, 38 [24–75] vs. 24 [19–41] days, 37 [22–75]vs. 25 [20–43] days *p* < 0.001, *p* < 0.001, *p* < 0.01, respectively; see Table 3). In line with the longer hospital stay and higher complication rates, the estimated median procedural costs were considerably higher in cases with preoperative myosteatosis based on all three criteria (68 [47–106] vs. 49 [36–63] TEuro, 60 [47–99] vs. 49 [37–66] TEuro, 68 [47–107 vs. 49 [36–64] TEuro, *p* < 0.001, for all, Table 3). Interestingly, among the tested myosteatosis selection criteria, only L3Muscle-RA and L3IMAC were suitable to identify patients at risk for EAD (36% vs. 24%, *p* = 0.016 for both L3Muscle-RA and L3IMAC, Table 3). More data on perioperative outcomes are outlined in Table 3.

**Figure 3:**
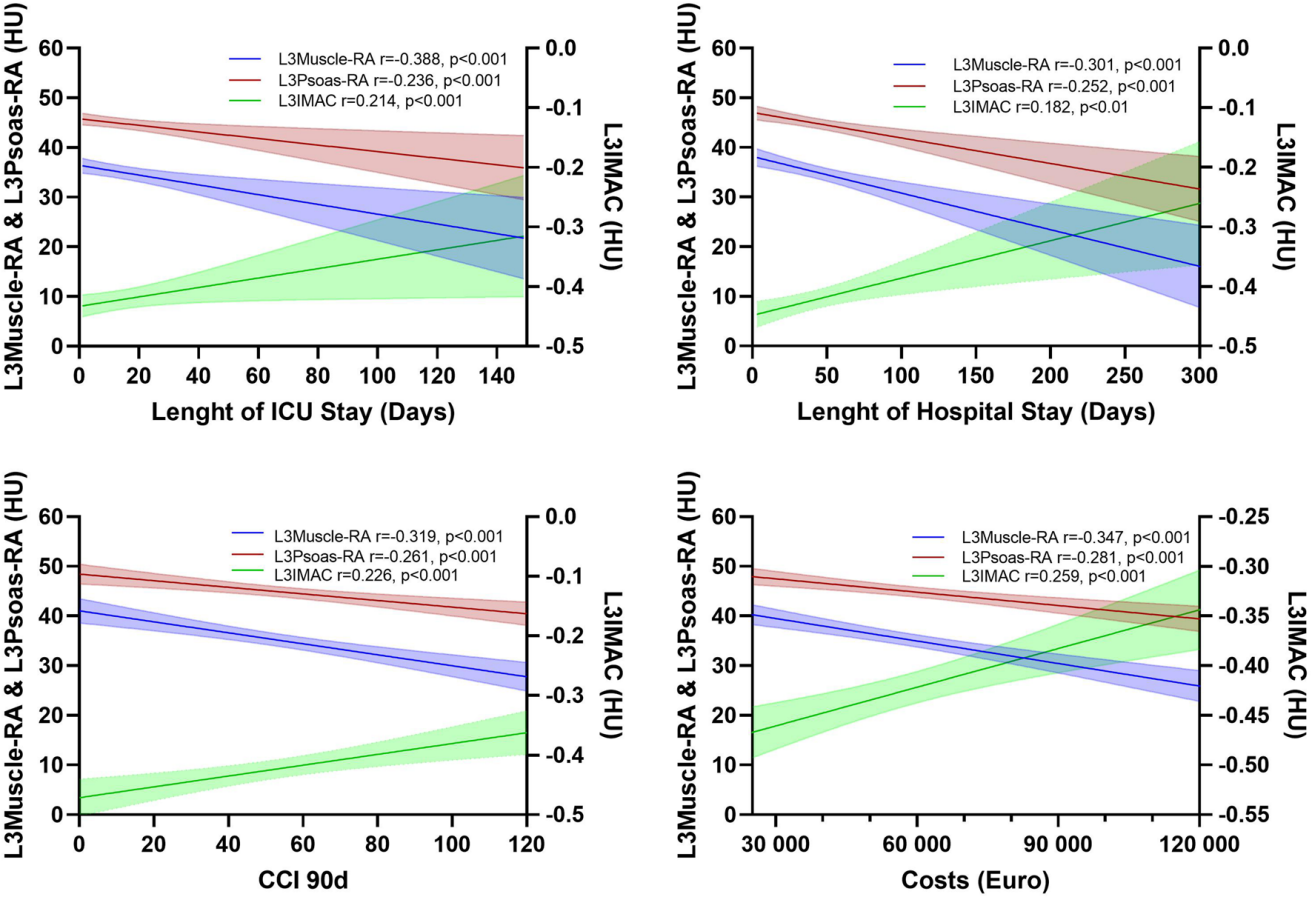
Correlation analysis between perioperative outcome and body composition selection criteria. Spearman correlation plots including ± 95% confidence interval for the association between L3-Muscle-RA; L3Psoas-RA; L3IMAC and length of ICU stay (**A**), length of Hospital Stay (**B**), CCI 90d (**C**), Costs (**D**). *Abbreviations used*: L3Muscle-RA: lumbar 3 muscle radiation attenuation, HU: Hounsfield Units, L3Psoas-RA: lumbar 3 Psoas radiation attenuation, L3IMAC: lumbar 3 intramuscular adipose tissue content, ICU: intensive care unit, CCI: Comprehensive Complication Index.

The association between perioperative outcomes and L3Muscle-RA, L3Psoas-RA, L3IMAC were studied further using the Spearman’s correlation coefficient and corresponding correlations plots (Fig. 3). In accordance with the above-described findings, a weak to moderate but significant association was observed between all of tested myosteatosis selection criteria and the length of ICU stay (L3Muscle-RA: r = − 0.338, *p* < 0.001; L3Psoas-RA: r = − 0.236, *p* < 0.001; L3IMAC: r = 0.214, *p* < 0.001, Fig. 3) and total in-hospital stay (L3Muscle-RA: r = − 0.301, *p* < 0.001; L3Psoas-RA: r = − 0.252, *p* < 0.001; L3IMAC: r = 0.182, *p* < 0.001, Fig. 3). Likewise, the 90-days CCI score and procedural costs were associated with all of the 3 parameters (CCI: L3Muscle-RA: r = − 0.319, *p* < 0.001; L3Psoas-RA: r = − 0.261, *p* < 0.001; L3IMAC: r = 0.226, *p* < 0.001, Fig. 3; Costs: L3Muscle-RA: r = − 0.347, *p* < 0.001; L3Psoas-RA: r = − 0.281, *p* < 0.001; L3IMAC: r = 0.259, *p* < 0.001, Fig. 3). In this analysis L3Muscle-RA showed slightly superior results and a stronger association with outcomes compared to L3Psoas-RA and especially when compared to L3IMAC (Fig. 3).

Further, L3Muscle-RA and L3IMAC showed a significant association with Body Mass Index (BMI) as a conventional antrophometric parameter, while no correlation could be found between L3Psoas-RA and BMI (L3Muscle-RA: r = − 0.213, *p* < 0.01; L3Psoas-RA: r = − 0.043, *p* = 0.483; L3IMAC: r = 0.414, *p* < 0.001, supplementary Fig. 2). Interestingly, BMI was not associated with perioperative outcome including 90-days CCI score and costs (CCI: r = 0.068, *p* = 0.274; Costs: r = 0.078, *p* = 0.213, supplementary Fig. 2).

**Figure 4:**
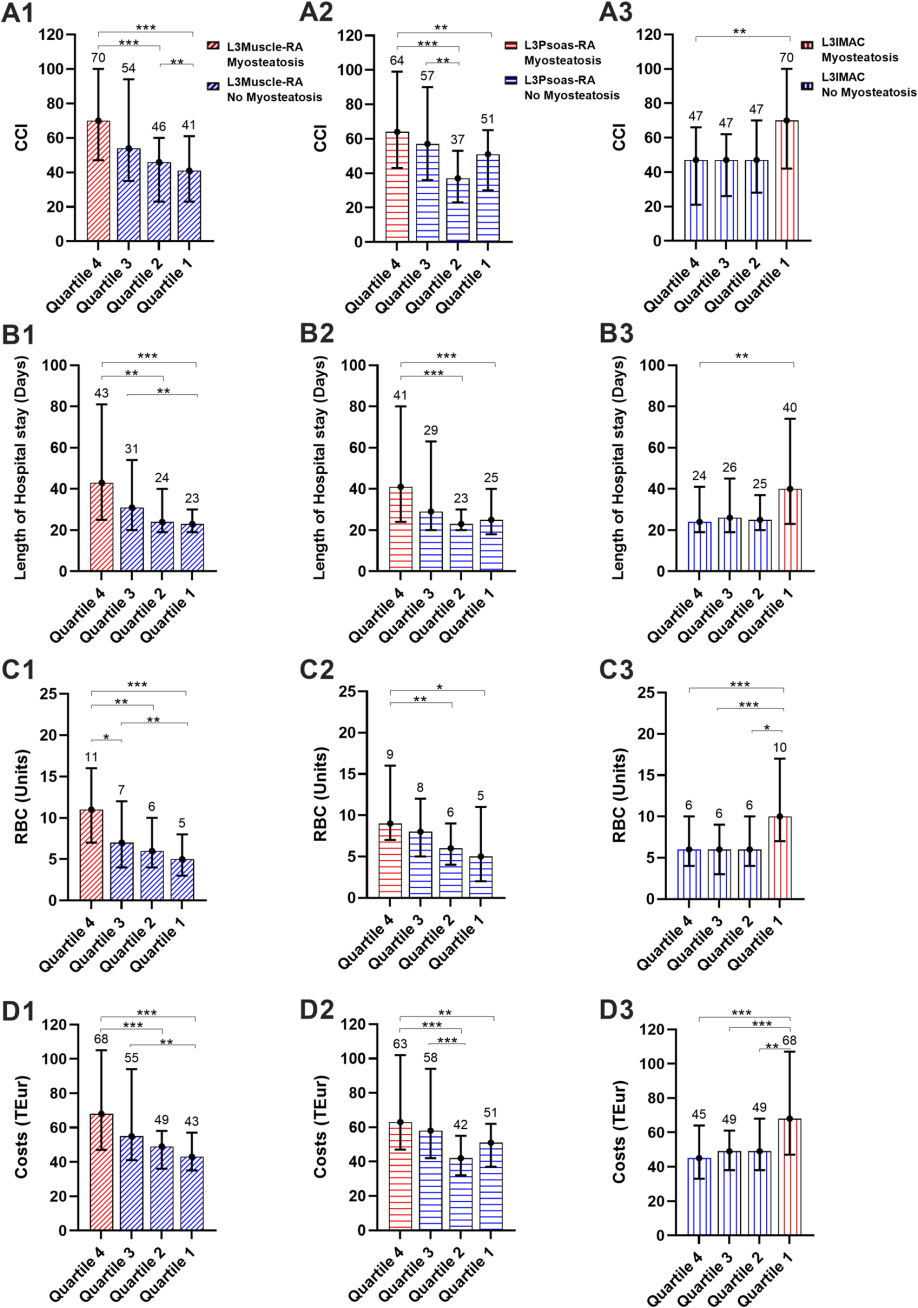
Quartile-based analysis of transfusion requirements, length of hospital stay, postoperative complications, and costs. Intraoperative transfusion of red blood cell (RBC) units according to L3Muscle-RA (**A1**), L3Psoas-RA (**A2**), L3IMAC (**A3**); length of hospitalization according to L3Muscle-RA (**B1**), L3Psoas-RA (**B2**), L3IMAC (**B3**); Comprehensive Complication Index (CCI) according to L3Muscle-RA (**C1**), L3Psoas-RA (**C2**), L3IMAC (**C3**), and procedural costs according to L3Muscle-RA (**D1**), L3Psoas-RA (**D2**), L3IMAC (**D3**). (median and IQR), *p < 0.05, **p < 0.01, ***p < 0.001, one-way ANOVA and Bonferroni post-hoc test, n = 66, 66, 66, 66, respectively) *Abbreviations used*: L3Muscle-RA: lumbar 3 muscle radiation attenuation, L3Psoas-RA: lumbar 3 Psoas radiation attenuation, L3IMAC: lumbar 3 intramuscular adipose tissue content.

Next, OLT recipients have been divided into quartiles, based on the distribution of L3Muscle-RA, L3Psoas-RA, L3IMAC values over the patient cohort (Fig. 4). This analysis, led to the observation that while L3Muscle-RA resulted in a satisfactory stratification of our patients even in individuals with superior muscle quality (Q1–Q3), L3Psoas and L3IMAC were not able differentiate in terms of outcomes between the patient quartiles with higher RA values (Q1–Q3, 75% of the cohort, Fig. 4). This was manifested in a gradual decrease in transfusion needs (Quartile 4 vs. 1 L3Muscle-RA: 11 [7–16] vs. 5 [3–8], *p* < 0.001; L3Psoas-RA: 9 [7–15] vs. 5 [2–11], *p* < 0.05; L3IMAC: 6 [4–10] vs. 10 [7–17], *p* < 0.001, Fig. 4), length of hospital stay (Quartile 4 vs. 1 L3Muscle-RA: 43 [25–81] vs. 23 [19–30] days, *p* < 0.001; L3Psoas-RA 41 [24–80] vs. 25 [18–40] days, *p* < 0.001; L3IMAC: 24 [19–41] vs. 40 [23–74] days, *p* < 0.01, Fig. 4), CCI (Quartile 4 vs. 1 L3Muscle-RA: 70 [47–100] vs 41 [23–61], *p* < 0.001; L3Psoas-RA: 64 [43–99] vs. 51 [30–65], *p* < 0.01; L3IMAC: 47 [21–66] vs. 70 [42–100], *p* < 0.001, Fig. 4) and costs (Quartile 4 vs. 1 L3Muscle-RA: 68 [47–106] vs. 43 [35–57] TEur, *p* < 0.001; L3Psoas-RA: 63 [47–102] vs. 51 [37–62] TEur, *p* < 0.01; L3IMAC: 45 [33–64] vs. 68 [47–107] TEur, *p* < 0.001, Fig. 4) when L3Muscle-RA was used. However, this gradual or step-wise pattern was not observed when L3Psoas-RA or L3IMAC were used to stratify our cohort (see e.g. Fig. 4 B3 or B2). Based on this, despite their relatively good performance in the identification of high-risk individuals in the group analyses, L3Psoas-RA and L3IMAC were not able to differentiate between patients with better muscle quality and less advanced myosteatosis (Fig. 4).

Analyzing the AUROCs, the best results in terms of the discriminative ability of the three tested parameters were obtained using 90-day mortality as outcome (Table 4). Here L3Muscle-RA and L3Psoas-RA showed satisfactory high AUROC values (> 0.7) with significant results and satisfactory model fit (L3muscle-RA: 0.762 *p* < 0.001; L3Psoas-RA: 0.751 *p* < 0.001; L3IMAC: 0.703 *p* = 0.077; Table 4). In this analysis, L3IMAC showed inferior performance compared to the other two parameters with either non-significant AUROC values due to broader confidence intervals or an insufficient model fit (Table 4).

**Table 4:** AUROC analysis and goodness-of-fit testing for the various myosteatosis selection criteria based on major complications (≥ CD3b), 90-day mortality and early allograft dysfunction.

Myosteatosis L3Muscle-RA		AUC	SE	95% CI	*p* value	Chi^2^*	*p* value^#^
90-day ≥ CD3b complications^a^	All	0.651	0.034	0.585–0.718	0.000	4.364	0.823
	Female	0.664	0.057	0.551–0.777	0.008	7.329	0.502
	Male	0.646	0.042	0.564–0.728	0.001	5.444	0.709
90-day mortality	All	0.762	0.047	0.699–0.855	0.000	5.923	0.656
	Female	0.742	0.091	0.562–0.921	0.025	5.000	0.758
	Male	0.771	0.055	0.664–0.878	0.002	7.796	0.454
Early allograft dysfunction^b^	All	0.574	0.042	0.493–0.656	0.066	3.464	0.902
	Female	0.621	0.740	0.476–0.766	0.092	4.723	0.694
	Male	0.564	0.050	0.461–0.662	0.188	2.157	0.976
**Myosteatosis L3Psoas-RA**							
90-day ≥ CD3b complications	All	0.621	0.035	0.553–0.688	0.001	11.172	0.192
	Female	0.653	0.058	0.538–0.767	0.014	13.727	0.089
	Male	0.609	0.043	0.524–0.693	0.014	12.813	0.118
90-day mortality	All	0.751	0.048	0.657–0.845	0.000	3.654	0.887
	Female	0.758	0.070	0.622–0.895	0.017	7.710	0.462
	Male	0.744	0.067	0.612–0.875	0.005	3.756	0.878
Early allograft dysfunction	All	0.476	0.044	0.391–0.562	0.558	11.406	0.180
	Female	0.535	0.078	0.382–0.688	0.630	6.247	0.511
	Male	0.453	0.053	0.350–0.556	0.335	9.229	0.323
**Myosteatosis L3IMAC**							
90-day ≥ CD3b complications	All	0.622	0.035	0.554–0.690	0.001	16.308	0.038
	Female	0.603	0.061	0.484–0.722	0.096	7.616	0.472
	Male	0.632	0.042	0.548–0.715	0.003	9.002	0.342
90-day mortality	All	0.703	0.064	0.577–0.830	0.077	8.478	0.388
	Female	0.681	0.118	0.458–0.904	0.114	7.111	0.525
	Male	0.724	0.076	0.575–0.873	0.076	3.965	0.860
Early allograft dysfunction	All	0.571	0.041	0.491–0.651	0.077	2.384	0.967
	Female	0.633	0.069	0.497–0.768	0.065	6.901	0.439
	Male	0.544	0.050	0.446–0.642	0.369	2.650	0.954

*Hosmer–Lemeshow Chi^2^ # in case of a *p* value of < 0.05 the test rejects the null hypothesis of an adequate fit. ^a^Based on Clavien et al.^27^. ^b^Based on Olthoff et al.^23^. *Abbreviations used*: L3Muscle-RA: lumbar 3 muscle radiation attenuation, AUC: Area under the curve, SE: standard error, CI: Confidence Interval, CD: Clavien–Dindo classification, L3Psoas-RA: lumbar 3 psoas radiation attenuation, L3IMAC: lumbar 3 intramuscular adipose tissue content.

Finally, univariable logistic regression analyses showed a significant association of pre-transplant Child–Pugh Score, labMELD, pre-transplant ICU stay, warm ischemic time, intraoperative transfusion of RBC units and myosteatosis with major postoperative morbidity (CD ≥ 3b) (Table 5). No major difference was observed in terms of odds-ratios between the various myosteatosis selection criteria (L3muscle-RA: OR 3.175 95%CI 1.721–5.856, *p* < 0.001; L3psoas-RA: OR 2.625 95%CI 1.445–4.770, *p* = 0.002; L3IMAC: OR 3.072 95%CI 1.644–5.741, *p* < 0.001).

**Table 5:** Uni- and multivariable logistic regression analysis for 90-days major morbidity (Clavien–Dindo ≥ 3b).

	Major complications (CD ≥ 3b)^1^ n = 136	No- / minor complications (CD1-3a)^1^ n = 128	Univariable analysis		Multivariable analysis	
			Odds-ratio (95% Confidence Interval)	*p value	Odds-ratio (95% Confidence Interval)	*p* value
Donor age ≥ 60 years	56 (21)	52 (20)	1.009 (0.616–1.652)	0.972		
Donor BMI ≥ 25	103 (39)	97 (37)	0.962 (0.542–1.7708)	0.896		
Donor sex Male	79 (30)	61 (23)	1.503 (0.922–2.452)	0.102		
Pre-transplant Child–Pugh Score ≥ 7	92 (35)	66 (25)	1.945 (1.176–3.217)	0.010	0.825 (0.435–1.565)	0.556
ECD^**a**^ Yes	87 (33)	83 (31)	0.939 (0.564–1.563)	0.939		
Recipient age ≥ 60 years	48 (18)	42 (16)	1.103 (0.662–1.840)	0.706		
Recipient BMI ≥ 25	98 (37)	78 (30)	1.630 (0.967–2.747)	0.067		
Recipient sex Male	89 (34)	84 (32)	0.967 (0.579–1.617)	0.899		
Pre-transplant labMELD ≥ 25	56 (21)	23 (9)	3.174 (1.801–5.597)	0.000	2.529 (1.054–6.046)	0.038
Recipient pre-transplant ICU Yes	44 (17)	17 (6)	3.100 (1.659–5.792)	0.000	1.072 (0.420–2.738)	0.884
Recipient pre-transplant abdominal surgery Yes	52 (20)	40 (15)	1.347 (0.808–2.245)	0.253		
Recipient pre-transplant encephalopathy Yes	58 (22)	42 (16)	1.506 (0.911–2.492)	0.111		
Cold ischemic time ≥ 480 (min)	74 (28)	64 (24)	1.216 (0.744–1.987)	0.435		
Warm ischemic time ≥ 45 min	66 (25)	75 (29)	0.653 (0.398–1.073)	0.088	0.616 (0.355–1.067)	0.084
Intra-operative red blood cell transfusions ≥ 15 units	35 (23)	5 (2)	8.400 (3.172–22.245)	0.000	8.571 (2.850–26.010)	0.000
Myosteatosis Yes: L3Muscle-RA	47 (18)	18 (7)	3.175 (1.721–5.856)	0.000	2.158 (1.098–4.245)	0.026^**#**^
L3Psoas-RA	45 (17)	20 (8)	2.625 (1.445–4.770)	0.002	1.962 (1.014–3.795)	0.045^**#**^
L3IMAC	46 (17)	19 (7)	3.072 (1.644–5.741)	0.000	2.021 (1.007–4.056)	0.048^**#**^

Values were given as numbers and (per cent). Results of the logistic regression were given as odds-ratios with 95% confidence interval. *Factors showing a *p* value < 0.1 in the univariable analysis were included in the multivariable logistic regression model. Only significant results are shown. ^#^To avoid a multicollinearity effect due to the inclusion of L3Muscle-RA, L3Psoas-RA, L3IMAC, the multivariable analyses were repeated for each of the three variables. ^a^Based on the German Medical Chamber Guidelines^31^.

*Abbreviations used*: BMI: body mass index, ECD: extended criteria donor allografts, MELD: model for end-stage liver disease, ICU: intensive care unit, L3Muscle-RA: lumbar 3 muscle radiation attenuation L3Psoas-RA: lumbar 3 Psoas radiation attenuation, L3IMAC: lumbar 3 intramuscular adipose tissue content.

In the multivariable analysis, labMELD (OR 2.529 95%CI 1.054–6.046, *p* = 0.038), intra-operative RBC transfusion (OR 8.571 95%CI 2.850–26.010, *p* < 0.001) and myosteatosis (L3muscle-RA: OR 2.158 95%CI 1.098–4.245, *p* = 0.026, L3Psoas-RA: OR 1.962 95%CI 1.014–3.795, *p* = 0.045, L3IMAC: OR 2.021 95%CI 1.007–4.056, *p* = 0.048) have been identified as independent predictors of major morbidity following OLT and demonstrated statistically significant results with meaningful odds ratios (Table 5).

### Long-term graft- and patient survival

Patients who died within the first 90 days following OLT (n = 20) were excluded from this analysis to avoid the strong confounding effects of BC on short term outcomes. The median length of follow up for the patient cohort was 70 months (without 90-day mortality). When early mortality was excluded, alterations of muscle quality had no significant effects on long-term graft and patient survival. Neither the probability of long-term graft survival (L3Muscle-RA: 1-year: 88% vs. 90%, 3-years: 84% vs. 84%, 5-years: 84% vs. 78%, *p* = 0.542; L3Psoas-RA: 1-year: 87% vs. 90%, 3-years: 83% vs. 84%, 5-years 80% vs. 79%, *p* = 0.961; IMAC: 1-year: 87% vs. 90%, 3-years: 82% vs. 84%, 5-years 79% vs. 79%, *p* = 0.841; Supp. Figure 1) nor the probability of long-term patient survival differed significantly in patients with myosteatosis compared to patients with normal muscle quality (L3Muscle-RA: 1-year: 88% vs. 94%, 3-years: 84% vs. 88%, 5-years: 84% vs. 83%, *p* = 0.918; L3Psoas-RA: 1-year: 87% vs. 94%, 3-years: 83% vs. 88%, 5-years: 80% vs. 84%, *p* = 0.402; IMAC: 1-year: 87% vs. 94%, 3-years: 82% vs. 88%, 5-years: 79% vs. 84%, *p* = 0.338; Supp. Figure 1).

## Discussion

This study provides insights into the performance of various frequently adopted selection criteria of muscle radiation attenuation and myosteatosis in predicting short- and long-term outcomes following deceased donor liver transplantation. Although, all three parameters showed an overall satisfactory performance in predicting perioperative morbidity and mortality, L3Muscle-RA was superior in the quartile based, correlation, and AUROC analyses. Neither of the used myosteatosis selection criteria was able to identify patients at risk for inferior long-term graft and patient outcomes, which is in line with previous findings showing that the strong prognostic value of myosteatosis seems to be particularly important in the early postoperative period^4,5^.

This study builds on the limited but continuously accumulating body of published evidence that BC and especially sarcopenia and myosteatosis are associated with worse clinical outcomes in patients with ESLD^32^. While previous reports provide ample evidence on the association between sarcopenia and outcomes^2,33^, only a handful of recent studies have suggested a potential value of myosteatosis in the setting of liver transplantation^4,12^. Even in case of these sporadic publications, there is a large heterogeneity concerning patient cohorts and the used selection criteria to define myosteatosis^12^. The lack of an international consensus on methodical definitions complicates the interpretation of these findings and results in inconclusive systematic reviews and meta-analyses^33^. This may ultimately impede the translation of BC assessment into clinical practice guidelines and international recommendations.

Malnutrition and consequential alteration in BC can be assessed with a broad variety of screening tools which have been validated in the past in various patient cohorts^7,34,35^. Although, CT-based image analysis and quantification of muscle mass (morphological aspect of sarcopenia) and muscle quality (myosteatosis) are considered to be the gold standard in patients with liver disease, a number of research groups have introduced various selection criteria and cutoff values to assess BC and identify patients with clinically relevant BC alterations^9,12,32^. Not only the mean attenuation values of the entire lumbar skeletal muscle area (L3Muscle-RA in our present study) but also the total psoas density (L3Psoas-RA in our present study) are frequently used by various authors to characterize myosteatosis in patients with liver disease^10,36^. In a recent study by Kalafateli et al., they recommended the bilateral psoas attenuation to characterize myosteatosis^37^. Based on these, the central and deep location of the psoas muscle, the more simple and precise identification of its exact borders would facilitate a precise image analysis and segmentation. Furthermore, the density and form of the psoas muscle are presumably less influenced by abdominal distension and disease-related water retention compared to other abdominal muscle components (e.g. ventral abdominal musculature)^37,38^.

Besides L3Muscle-RA and L3Psoas-RA, the lumbar multifidus muscle / subcutaneous fat tissue attenuation ratio, known as IMAC (L3IMAC in our present study), was used in multiple Japanese studies to determine myosteatosis^5,12,39^. In contrast to the absolute RA values, this novel parameter holds promise to reduce the variation between individual CT scans and patients, leading to an improved identification of clinically significant alterations^5,12^. A higher IMAC indicates an increased muscular adipose tissue content, thus a lower muscle quality^5,12^.

In our present report, the presence of myosteatosis, defined by the sex-specific quartile-based cutoff values for L3Muscle-RA, L3Psoas-RA, and L3IMAC, was significantly associated with inferior perioperative outcomes. Patients with myosteatosis presented with significantly increased morbidity and mortality (increased major complication rates ≥ CD3b and cumulative CCI) over the first 90 days following OLT and showed higher intraoperative transfusion needs and longer stay on the ICU and in hospital. This inferior perioperative outcome was manifested in increased costs over the first 3 months. Although, there were no major differences in the performance of the three analysed selection criteria for recipient myosteatosis in terms of perioperative outcomes in our group analysis, L3Psoas-RA and IMAC seemed to be slightly inferior compared to L3Muscle-RA in the identification of patients at risk for EAD in our group analysis (Table 3). In our quartile-based, correlation and AUROC analyses, however, L3Muscle-RA showed a superior discriminative and diagnostic ability.

While several Japanese studies have explored the association of IMAC with the severity of non-alcoholic steatohepatitis (NASH) and the outcomes following LDLT^5,11,12,40^, and our group and others have extensively investigated L3Muscle-RA and L3Psoas-RA^1–4,33,37,41^, none of these previous reports attempted to compare various selection criteria for muscle RA and myosteatosis in a liver transplantation cohort. IMAC was first described by Kitajima et al. showing a relationship between an increasing IMAC and disease severity in NASH patients^11,40^. As the values of IMAC have improved over time following therapeutic intervention such as dietary changes and exercise, the authors proposed IMAC as a potentially valuable marker to non-invasively monitor therapeutic success in patients with chronic liver disease. However, IMAC has later also been adopted for the “non-NASH” setting and the Kyoto group has investigated its role following LDLT. In their pioneering report by Hamaguchi et al., they have found a strong association (*p* < 0.01) between high IMAC values and post-transplant survival using living donors^5,12^. In our present study the probability of graft- and patient survival did not differ significantly over the follow-up period below and above the L3Muscle-RA, L3Psoas-RA, L3IMAC cutoffs. However, likewise in our findings, in the above-mentioned Japanese cohort a large number of the registered death events—thus the major difference in survival—occurred during the early post-LDLT phase with 90% of patients dying within the first year after LDLT^5,12^. Therefore, the lack of survival difference in our study may be attributed to our different statistical approach. To avoid the potentially interfering effects of early mortality we have excluded patients who died within the first 3 months after OLT (n = 20) from the analysis of long-term graft- and patient outcomes.

The findings of this study should be interpreted in the light of potential limitations. First, due to the retrospective nature of our analysis, the present study omitted any functional analysis of patient fitness and muscle strength which should be mentioned as an important limitation. Second, it is also necessary to consider whether the used L3Muscle-RA, L3Psoas-RA, and L3IMAC cutoffs used in our group analysis were adequate to identify patients at risk for inferior outcomes. Here we chose to use sex-specific cutoff values to identify patients belonging to the lower 25% in our cohort in terms of muscle quality according to the 3 different myosteatosis selection criteria. However, to reduce potential bias associated with this approach, we have also used further sophisticated and comprehensive statistical methods to analyze and report the diagnostic value and limitations of these three parameters from various angles (AUROC analysis, quartile-based distribution and correlation analysis). Third, our analyzed patient cohort shows the general characteristics of a heterogeneous European OLT patient cohort which carries the risk of a certain selection bias and may led to the underrepresentation of various indications and patient subgroups (e.g. high-MELD patients or patients with NASH). Fourth, CT scans used for segmentation analysis were obtained preoperatively as part of the clinical routine at heterogeneous time points and analysed in a retrospective and uncontrolled fashion.

Notwithstanding these limitations, this report is one of the first comprehensive studies assessing and comparing the value and limitations of three different but frequently reported radiation attenuation-based selection criteria for myosteatosis, demonstrating a comparable performance and similar shortcomings for all three parameters in predicting short- and long-term outcomes following deceased donor OLT. L3Muscle-RA has performed slightly superior compared to L3Psoas-RA and L3IMAC (depicted e.g. by the prediction of EAD as well as in a better linear correlation with ICU and hospital stay, CCI and costs or by its superior performance in the quartile-based or AUROC analyses). Based on these promising results, an international consensus and standardization of selection criteria would be highly desirable to improve comparability and reproducibility of findings and facilitate rapid translation of BC-based and malnutritional scores into clinical risk-assessment and outcome prediction in the setting of OLT. Further studies are warranted not only just to provide an external validation for these findings but to test the prognostic robustness of myosteatosis in various highly selected patient cohorts using a multi-center setting with a sufficient sample size and statistical power. It might be of particular clinical interest to investigate the prognostic role of myosteatosis in severe morbidity and mortality using a larger set of selected high-MELD patients which was not possible in a statistically meaningful way in the present single-center study.

## Supplementary Information

Supplementary Figure 1.

Supplementary Figure 2.

## Data Availability

All relevant data were reported within the manuscript and the supplementary files. Further supporting data will be provided upon written request addressed to the corresponding author.

## Abbreviations

ALF: Acute liver failure
ANOVA: Analysis of Variance
AUC: Area Under the Curve
AUROC: Area Under the Receiver Operating Characteristics Curve
BAR: Balance of Risk
BC: Body Composition
BMI: Body Mass Index
CCI: Comprehensive Complication Index
CD: Clavien–Dindo classification
CI: Confidence Interval
CT: Computed Tomography
CVA: Cerebrovascular Accident
EAD: Early Allograft Dysfunction
EASL: European Association for the Study of the Liver
ECD: Extended Criteria Donor
FFP units: Fresh Frozen Plasma units
GCP: Good Clinical Practice
HU: Hounsfield unit
ICH: International Conference on Harmonisation
ICU: Intensive Care Unit
IMAC: Intramuscular Adipose Tissue Content index
L3: Third lumbar level
MELD: Model of End-stage Liver Disease
OLT: Orthotopic Liver Transplantation
OR: Odds-ratio
PACS: Picture Archiving and Communication System
PBC: Primary Biliary Cholangitis
POD: Postoperative day
PSC: Primary Sclerosing Cholangitis
RA: Radiation attenuation
RBC units: Red Blood Cell units
SE: Standard error
SOFT: Survival outcomes following liver transplantation
TEur: Thousand Euros
UH-RWTH: University Hospital of the RWTH University
