# Supplementary material for: Various myosteatosis selection criteria and their value in the assessment of short- and long-term outcomes following liver transplantation
Source: Sci Rep. 2021 Jun 28;11:13368. doi: 10.1038/s41598-021-92798-5 (PMC8239038; doi:10.1038/s41598-021-92798-5)
Supplement: Supplementary file 1 — Supplementary Figure 1. [file 41598_2021_92798_MOESM1_ESM.docx]

**
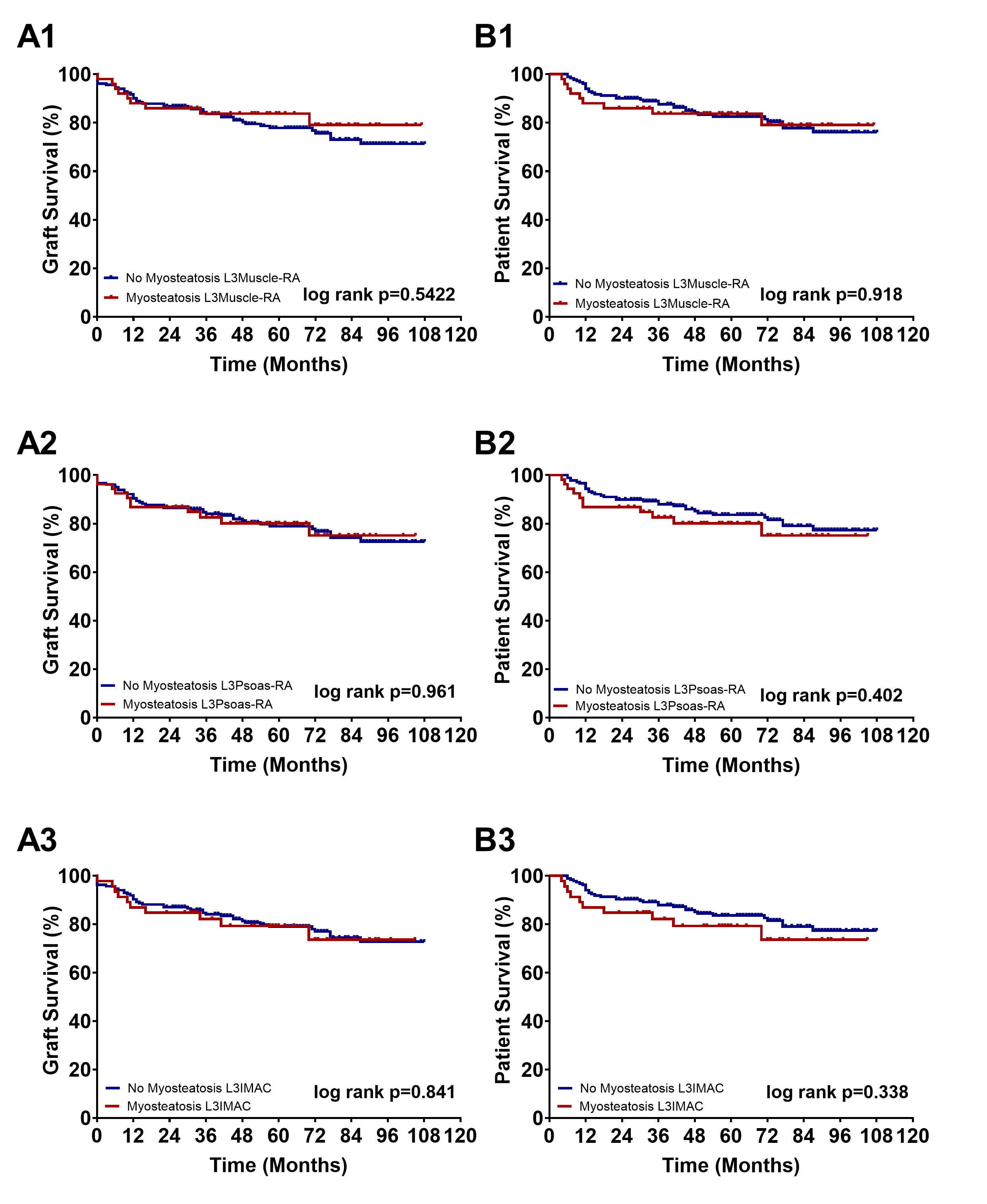
**

**Supplementary Figure 1:** Graft and patient survival stratified by myosteatosis after excluding 90-day mortality

To show the long-term effects of myosteatosis on graft and patient survival, patients who died within 90 days of transplantation (n=20) were excluded. (A1) Graft and (B1) patient survival by No Myosteatosis 84% vs. Myosteatosis 78% according to L3Muscle-RA. (A2) Graft and (B2) patient survival by No Myosteatosis 80% vs. Myosteatosis 79% according to L3Psoas-RA. (A3) Graft and (B3) patient survival by No Myosteatosis 79% vs. Myosteatosis 79% according to L3IMAC. Abbreviations used: L3Muscle-RA: lumbar 3 muscle radiation attenuation, L3Psoas-RA: lumbar 3 Psoas radiation attenuation, L3IMAC: lumbar 3 intramuscular adipose tissue content.
