# Supplementary material for: Various myosteatosis selection criteria and their value in the assessment of short- and long-term outcomes following liver transplantation
Source: Sci Rep. 2021 Jun 28;11:13368. doi: 10.1038/s41598-021-92798-5 (PMC8239038; doi:10.1038/s41598-021-92798-5)
Supplement: Supplementary file 2 — Supplementary Figure 2. [file 41598_2021_92798_MOESM2_ESM.docx]

**
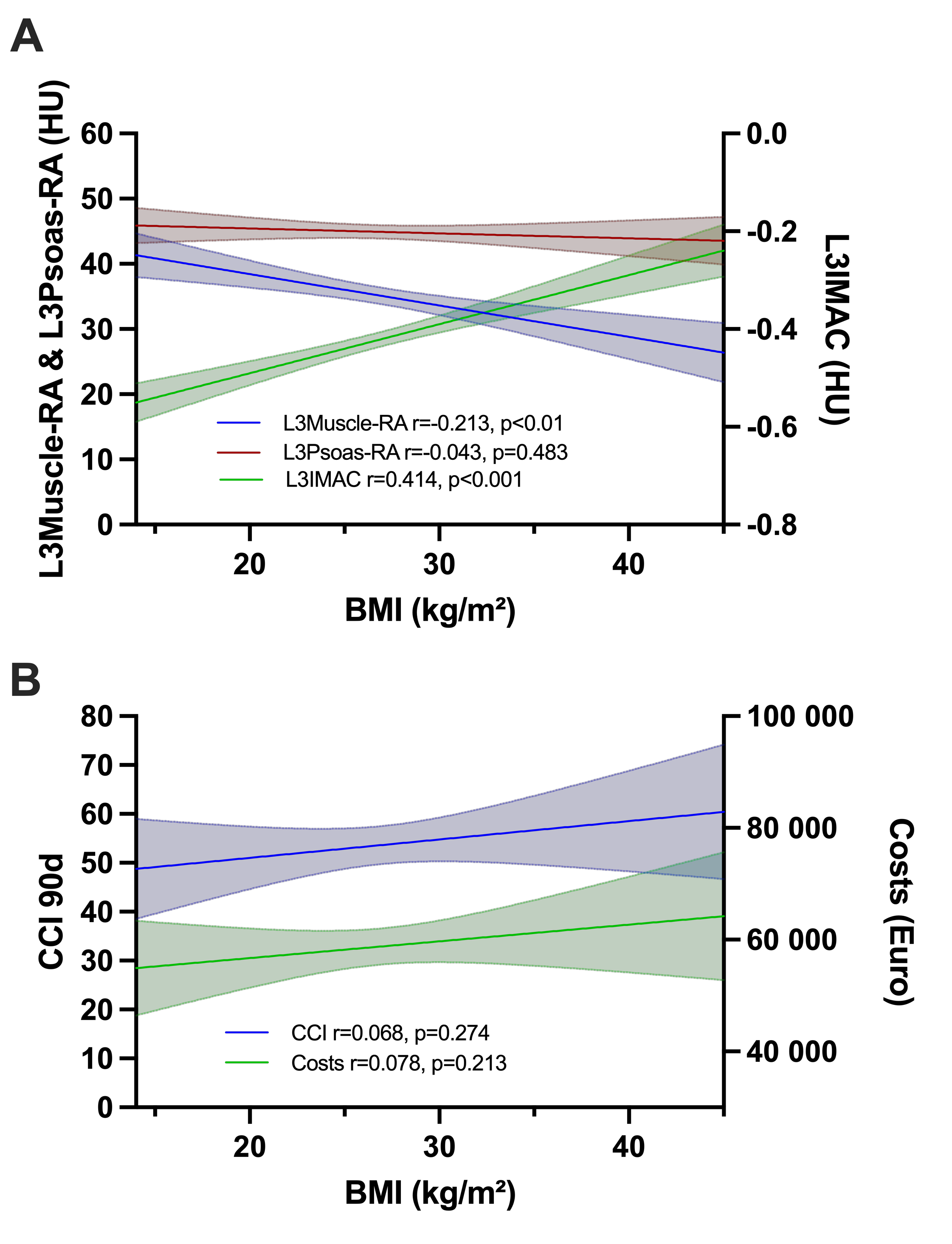
**

**Supplementary Figure 2:** Correlation analysis between body composition selection criteria, perioperative outcome and Body Mass Index

Spearman correlation plots including ±95% confidence interval for the association between BMI and L3-Muscle-RA; L3Psoas-RA; L3IMAC (A) and perioperative outcome including 90-day CCI and estimated procedural costs.

Abbreviations used: L3Muscle-RA: lumbar 3 muscle radiation attenuation, HU: Hounsfield Units, L3Psoas-RA: lumbar 3 Psoas radiation attenuation, L3IMAC: lumbar 3 intramuscular adipose tissue content, BMI: Body Mass Index, CCI: Comprehensive Complication Index
